# A rare case of invasive non-typeable *Haemophilus influenzae* spondylodiscitis and periprosthetic joint infection

**DOI:** 10.5194/jbji-6-207-2021

**Published:** 2021-06-02

**Authors:** Kevin Sermet, François Demaeght, Isabelle Alcaraz, Nathalie Viget, Julie Dauenhauer, Eric Senneville, Olivier Robineau

**Affiliations:** 1 Gustave Dron Hospital, 59200, Tourcoing, France; 2 Department of Infectious Disease, University of Lille, 59000, Lille, France

## Abstract

A non-typeable *Haemophilus influenzae* (NTHi) was responsible for an invasive infection including bacteremia, spondylodiscitis with epidural abscess, and periprosthetic hip infection in a 79-year-old woman, triggered by a superinfected ethmo-orbital mucocele. Surgical drainage and antibiotic therapy allowed recovery. PET-scan full cartography of NTHi infection dissemination enabled the discovery of spondylodiscitis. This rare cause of spondylodiscitis and periprosthetic joint infection suggests a complete work-up is unavoidable.

## 1 Introduction

*Haemophilus influenzae* (Hi) is a nasopharynx-commensal Gram-negative bacillus mostly known for its encapsulated species, responsible for either non-severe (e.g., uncomplicated otitis media, sinusitis, conjunctivitis) or severe infections (e.g., pneumonia, bacteremia, meningitis), and is targeted by a conjugated vaccine against its polysaccharide capsule (Bakaletz and Novotny, 2018). Non-typeable Hi (NTHi) emerges in vaccine-covered areas as the most prevalent *Haemophilus* species, causing non-severe infections of the respiratory tract (Dworkin et al., 2007) because vaccines are not commercialized yet for these non-encapsulated strains. Apart from neonatal sepsis and meningitis, only a few invasive infections are reported (Dworkin et al., 2007; ABCs report of CDC, 2019). Infections in adults mainly occur with a chronic respiratory disease or immune deficiency history, and NTHi is even more uncommon in osteomyelitis (Kim et al., 2011).

Here we report a rare case of an invasive NTHi infection in a 79-year-old patient including a lumbar spondylodiscitis with epidural abscess and a periprosthetic joint infection (PJI).

**Figure 1:**
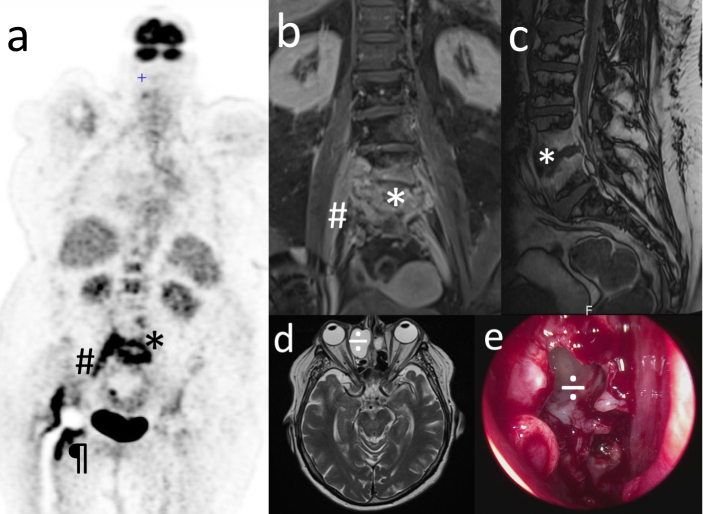
Non-typeable *Haemophilus influenzae* invasive spondylodiscitis started from a bacteremia of the ENT entry site and shows an unreported mechanism of dissemination. **(a)** PET scan; other images show a continuous fixation along the iliopsoas muscle; **(b, c)** spine and pelvic T1-weighted gadolinium-enhanced MRI: ${}^{*}$ L4–L5 spondylodiscitis with epidural abscess; # right medial iliopsoas infiltration; ¶ hip prosthesis infection. **(d)** Sinus T2-weighted MRI and **(e)** peri-operative view of endo-nasal surgery after opening the right ethmo-orbital mucocele: ÷ mucocele revealed a clear mucoid content that underwent *Haemophilus influenzae*-specific PCR, positive for a non-typeable strain. **(a)**, **(b)**, **(c)**, and **(d)** are courtesy of the Radiology Department of Gustave Dron Hospital.

## 2 Case description

A 79-year-old woman sought treatment at the emergency room for fever and fast-onset hip pain. She had full autonomy, and her medical history included well-controlled non-insulin-dependent diabetes, hypertension, atrial fibrillation, and a right hip fracture treated by total joint arthroplasty 10 years ago. Interrogation revealed no uptake of non-steroid anti-inflammatories or immunosuppressors and no alcohol abuse. Clinical examination revealed fever (38.5 ${}^{\circ }$C) and mild right hip pain without limitation of the joint range of motion without erythema or swelling and with no sign of other arthritis. Cardiopulmonary auscultation, ear, nose and throat (ENT) area, neurological testing, and the rest of the examination were normal. Blood tests showed an increased neutrophil count (11.300 mm${}^{-3}$), decreased lymphocyte count (600 mm${}^{-3}$), and increased CRP (90 mg L${}^{-1}$) without renal or hepatic dysfunction.

The patient was first admitted to the orthopedic department and later on to the infectious diseases unit of a reference center since blood cultures were positive for NTHi after matrix-assisted laser desorption ionization–time of flight (MALDI-TOF) analysis on bacterial isolates. The strain showed susceptibility to ciprofloxacin with a minimum inhibitory concentration (MIC) of 0.380 mg L${}^{-1}$, amoxicillin (MIC of 0.750 mg L${}^{-1}$), and cefotaxime (0.008 mg L${}^{-1}$). Two grams of cefotaxime every 8 h was initiated intravenously, with a good clinical and biological response. Joint aspiration of the right hip prosthesis was unfortunately performed after the initiation of cefotaxime therapy, with 5 mL of a cloudy liquid showing visually increased neutrophil count (without formula because of too many red cells) and sterile 14 d culture in solid and broth aerobic and anaerobic media. Polymerase chain reaction (PCR) could not performed because no liquid was kept after the patient transfer.

A plain X-ray of the right hip and tomodensitometry showed no sign of prosthetic infection or fracture. Transthoracic and transesophageal echocardiography ruled out endocarditis. Nevertheless, fluorine-18fluorodeoxyglucose positron emission tomography/computed tomography (PET) scan showed intense fixations of the L4–L5 vertebras, the right iliopsoas (continuously as shown in other images), and right periprosthetic tissues (Fig. 1a), consistent with magnetic resonance imaging (MRI), which showed an iliopsoas infiltration and epidural abscess-associated spondylodiscitis (Fig. 1b and c).

Advanced interview revealed that right-eye conjunctivitis had occurred 2 weeks before this episode. Sinus computerized tomography (CT) and MRI revealed a right ethmoido-orbital mucocele responsible for lysis of the medial orbital wall but not the anterior cranial fossa (Fig. 1d). To release the osteolytic pressure, allow bone reconstruction, and avoid further complications, naso-endoscopic surgery was performed (after a normal ophthalmologic examination was confirmed) through a mid-turbinate and opened mucocele (Fig. 1e). It released a clear mucoid content on which specific *Haemophilus* spp. PCR revealed a non-typeable strain. No sign of malignancy was found on pathologic analysis.

Surgery was delayed due to the altered general state at the time of transfer. Prosthetic hip replacement following the antibiotic treatment was thus decided on to ensure a fast rehabilitation after prolonged bed rest. The patient was treated with 12-week-duration antibiotic therapy of intravenous cefotaxime for 21 d followed by oral ciprofloxacin 750 mg twice a day. The patient displayed full recovery, and no adverse event was noted.

No additional immunodeficiency other than a mild lymphopenia was found: abdominal CT showed full hepato-splenic integrity, no Howell–Jolly bodies were found, and complement exploration, lymphocyte immunophenotyping, serum protein electrophoresis, quantitative immunoglobulin assay, and IgG subclasses showed no other abnormalities.

## 3 Discussion

NTHi emerges as the first type of invasive *Haemophilus* spp. infections and now exceeds Hi types b and f (Wan Sai Cheong et al., 2015). Nevertheless, they remain rare, all the more in adults. To our knowledge, it is the fourth reported NTHi-induced spondylodiscitis (Boulton et al., 2012; Personius and Camp, 1997; Van der Ploeg et al., 2008) and the first with epidural abscess and periprosthetic joint infection. This case is remarkable by its initial trigger, its severity, and the lack of patient immunosuppression.

The sinus imaging led to identification of the mucocele as a probable trigger of NTHi dissemination secondary to superinfection by a sporadic event. This suggests sinus imaging should be considered in every *Haemophilus* spp. invasive infection if no pneumonia is identified, as previously reported for *H. parainfluenzae* (Cobo et al., 2017), and the treatment of a chronic sinus or orbital condition may prevent resurgence in such cases.

The PET scan was crucial in this case because it spotlighted a continuous spine-to-hip septic channel possibly leaving an active spondylodiscitis disseminating down the iliopsoas, thus leading to a periprosthetic joint infection since its distal attachment is located on the trochanter minor with extreme proximity to the hip joint. PET scan appears to be useful in invasive *Haemophilus* spp. infection management, and we suggest that it may be performed in patients with *Haemophilus*-documented lumbar spondylodiscitis and a history of total hip replacement.

While NTHi infection is associated with an identified cause of B-cell impairment (mainly hemopathies) in 38 % of adult cases (Resman et al., 2011), our patient had well-controlled diabetes mellitus and mild lymphopenia. Age-related B-cell dysfunction might play a role in the resurgence of such infections, and even minor *Haemophilus* infections should not be underestimated because of its dissemination potential in the elderly.

This rare case report highlights that NTHi can be responsible for severe infections even without a patent immune disorder and that a complete exploration for an extension is required in these settings.

## Data Availability

Data underlying this work cannot be publicly published for patient confidentiality purposes.
